# Supplementary material for: Complexome profiling of the Chlamydomonas psb28 mutant reveals TEF5 as an early PSII assembly factor
Source: Plant Cell. 2025 Mar 17;37(6):koaf055. doi: 10.1093/plcell/koaf055 (PMC12164590; doi:10.1093/plcell/koaf055)
Supplement: koaf055_Supplementary_Data [file koaf055_supplementary_data.zip › psb28_interactive.zip!/plots/intensity/qualityPlots/peptides/AAA1_Cre08.g358526.t1.1.html]

### Cre08.g358526.t1.1

Trivial name: AAA1  
SeqCoverage [median]: 15.10  
Mass: 62.31 kDa  
  
 

| Sequence | MissedCleavage | Mass [Da] | wtIntensity | muIntensity | Score |
| --- | --- | --- | --- | --- | --- |
| DTKDVLVVTAPGSGAEIIPFLK | 1 | 2269.26 | 20536.22 | 19915.90 | 128.67 |
| DVLVVTAPGSGAEIIPFLK | 0 | 1925.09 | 71033.38 | 61737.97 | 190.37 |
| EMAYIPLEDEVK | 0 | 1435.69 | 0.00 | 8022.31 | 148.91 |
| EMAYIPLEDEVKTK | 1 | 1664.83 | 0.00 | 2809.81 | 68.00 |
| FAGPIAILR | 0 | 956.58 | 127627.21 | 437360.12 | 148.21 |
| FLGFEPITWAK | 0 | 1307.69 | 555.25 | 3823.58 | 110.14 |
| KFLGFEPITWAK | 1 | 1435.79 | 9826.50 | 0.00 | 80.62 |
| QFTALQEETGMYITGEK | 0 | 1944.91 | 0.00 | 6538.34 | 179.93 |
| QFTALQEETGMYITGEKPANK | 1 | 2355.14 | 112245.34 | 392382.71 | 233.59 |
| QFTALQEETGMYITGEKPANKSEK | 2 | 2699.31 | 0.00 | 4029.38 | 54.22 |
| YFSQVR | 0 | 798.40 | 134186.13 | 391094.31 | 196.43 |
|  |  |  | 476010.0 | 1327714.4 |  |
